# Red-fleshed apple flavonoid extract alleviates CCl_4_-induced liver injury in mice

**DOI:** 10.3389/fnut.2022.1098954

**Published:** 2023-01-18

**Authors:** Yizhou Chen, Yanbo Wang, Shenghui Jiang, Jihua Xu, Bin Wang, Xiaohong Sun, Yugang Zhang

**Affiliations:** ^1^College of Horticulture, Qingdao Agricultural University, Qingdao, China; ^2^Engineering Laboratory of Genetic Improvement of Horticultural Crops of Shandong Province, Qingdao Agricultural University, Qingdao, China; ^3^College of Life Sciences, Qingdao Agricultural University, Qingdao, China

**Keywords:** red-fleshed apple, flavonoid, liver injury, intestinal microorganisms, high-performance liquid chromatography

## Abstract

In recent years, the global incidence of liver damage has increased. Despite the many known health benefits of red-fleshed apple flavonoids, their potential liver-protective effects have not yet been investigated. In this study, we analyzed the composition of red-fleshed apple flavonoid extract (RAFE) by high-performance liquid chromatography (HPLC). We then induced liver damage in mice with carbon tetrachloride (CCl_4_) and performed interventions with RAFE to analyze its effect on liver damage, using bifendate as a positive control. The results showed that catechin was the most abundant flavonoid in ‘XJ4’ RAFE (49.346 mg/100 g). In liver-injured mice, the liver coefficients converged to normal levels following RAFE intervention. Moreover, RAFE significantly reduced the enzymatic activity levels of glutamic oxaloacetic transaminase (ALT), glutamic alanine transaminase (AST), and alkaline phosphatase (ALP) in mouse serum. Furthermore, RAFE significantly increased the content or enzyme activity level of total glutathione, total antioxidant capacity, and superoxide dismutase, and significantly decreased the content of malondialdehyde in the liver of mice. In parallel, we performed histopathological observations of mouse livers for each group. The results showed that RAFE restored the pathological changes caused by CCl_4_ around the central hepatic vein in mice and resulted in tightly bound hepatocytes. The recovery effect of RAFE was dose-dependent in the liver tissue. Regarding intestinal microorganisms, we found that RAFE restored the microbial diversity in liver-injured mice, with a similar microbial composition in the RAFE intervention group and normal group. RAFE reduced the ratio of *Firmicutes* to *Bacteroidetes*, increased the levels of probiotic bacteria, such as *Lactobacillus acidophilus*, and *Clostridium*, and reduced the levels of harmful bacteria, such as *Erysipelothrix Rosenbach.* Therefore, RAFE ameliorated CCl_4_-induced liver damage by modulating the abundance and composition of intestinal microorganisms in mice. In conclusion, RAFE alleviated CCl_4_-induced liver damage in mice, with H-RAFE (5 mg kg^–1^) significantly improving liver damage in mice but M-RAFE (1 mg kg^–1^) significantly improving the imbalance of intestinal microorganisms in mice. Our research suggests that RAFE could be employed for the adjuvant treatment and prevention of liver damage, and may have important applications in food and medicine.

## 1. Introduction

The liver is the largest digestive gland in the human body, participating in multiple vital functions, such as digestion, synthesis, storage, detoxification, and immunity (1, 2). However, the frequent abuse of alcohol, drugs, chemical additives, and food safety problems has led to annual increases in the incidence of liver injury. Persistent liver injury induces excessive proliferation and deposition of the extracellular matrix and gradually develops into liver fibrosis and cirrhosis, eventually leading to liver failure and even liver cancer (3). Liver injury is classified as acute or chronic according to the severity of the disease. Acute liver injury is a critical condition in humans, characterized by a rapid onset and significant damage, that can cause a large amount of liver tissue necrosis in a short period of time, as well as severe liver failure (4). Chronic liver injury has a complex pathogenesis and is characterized by abnormal proliferation and differentiation of hepatic stellate cells and cellular changes leading to massive deposition of extracellular matrix such as collagen, which ultimately results in abnormal intrahepatic tissue and fibroplasia (5). The main causes of liver injury are exposure to chemical agents (e.g., amoxicillin, ciprofloxacin, and oral contraceptives) and toxic compounds [e.g., alcohol, aflatoxin, and carbon tetrachloride (CCl_4_)]. Exposure of the liver to toxic substances leads to oxidative stress by generating large amounts of reactive oxygen species. Excess reactive oxygen species increase lipid peroxidation and cause oxidative injury to hepatocytes, which ultimately leads to fatty liver degeneration, chronic hepatitis, cirrhosis, and hepatocellular carcinoma (6–8). Currently, common treatments for liver injury include the use of specific detoxifying agents (e.g., N-acetylcysteine for acetaminophen toxicity), immunosuppressive therapy (e.g., corticosteroids for drug-induced hypersensitivity reactions or autoimmune injury), general hepatoprotective agents (e.g., ursodiol, silymarin, and glycopyrrolate) and liver transplantation (9). However, currently available treatments have various limitations (10). For example, specific antidotes can only treat specific cases of liver injury, and are virtually ineffective against general liver injury, immunosuppressive therapy is not always effective, and general hepatoprotective agents have little effect in the treatment of acute liver injury. Moreover, the drawbacks of liver transplantation include the limited availability of donor matches and the need for prolonged immunosuppressive therapy after transplantation (11). Therefore, it is crucial to find an effective treatment for liver injury that has a wide range of applications.

A study on CCl_4_-induced liver injury found that treatment with high concentrations of imatinib (a protein kinase inhibitor) effectively increased the non-protein sulfhydryl group content and superoxide dismutase (SOD) activity in the liver, but reduced myeloperoxidase activity in the liver to normal control values (12). However, serum levels of glutamic oxaloacetic transaminase (ALT), glutamic alanine transaminase (AST), and total bilirubin, as well as hepatic NADPH oxidase levels, were elevated after treatment with high concentrations of imatinib. These results indicate that treatment with high concentrations of imatinib did not protect the liver, but instead produced liver injury (12). Karimi et al. studied the protective effects of losartan (a receptor type I angiotensin inhibitor) combined with nilotinib (a multi-targeted tyrosine kinase inhibitor) on CCl_4_-induced liver fibrosis in rats, and found that this dual treatment had a synergistic effect. The treatment focused on the cytokines that are most important for improving liver fibrosis [transforming growth factor-β (TGF-β) and platelet-derived growth factor], and successfully reduced the expression of both cytokines. Although this treatment achieved the purpose of repairing liver injury, it also includes the side effects of some multi-targeted TK inhibitors, such as fatigue, rash, gastrointestinal symptoms, edema, neurological symptoms, etc., and is further inhibited by relatively high costs (13). Therefore, existing drugs for the treatment of CCl_4_-induced liver injury have certain disadvantages. Considering that natural active ingredients have few side effects, it is important to thoroughly explore the alleviation effect of such products on liver injury.

In recent years, several studies have confirmed the positive effects of plants and their natural products on the prevention and treatment of liver injury (14–16). Phenolic substances in plants are the most abundant and structurally diverse plant active substances, whose main role is alleviating oxidative injury, such as cancer, liver injury, and cardiovascular disease (17, 18). Moreover, polyphenols and flavonoids have the potential to protect against CCl_4_-induced liver injury in phenolics (19). Adewale and Abiodun reported that hibiscus polyphenol enrichment (HPE) exhibits protective and antioxidant effects against CCl_4_-induced liver injury (20). Specifically, elevated levels of malondialdehyde (MDA) were found in the livers of CCl_4_-treated animals, which represented an increase in hepatic lipid peroxidation and therefore hepatic injury; however, treatment with HPE significantly reduced lipid peroxidation levels, suggesting that its ability to protect against CCl_4_-induced liver injury may be attributed to its high antioxidant potential. Green tea polyphenols also inhibit oxidative injury and prevent CCl_4_-induced liver injury (21). Furthermore, total flavonoids extracted from *Bidens bipinnata* L. can improve acute liver injury in mice and liver fibrosis in rats by inhibiting oxidative stress. These results may be related to the inhibition of nuclear factor kappa B activation by these total flavonoids in hepatic stellate cells (22, 23). However, relatively few studies have investigated the role of polyphenols in apples on CCl_4_-induced liver injury, with red-fleshed apple variants being particularly rich in polyphenols, especially flavonoids.

Red-fleshed apples [*Malus sieversii* Roem. *f. niedzwetzkyana* (Dieck) Langenf.] are a variety of Xinjiang wild apples that are native to the Ili region of Xinjiang, China, and Kazakhstan in Central Asia, which have red or dark-red fruits, flowers, and leaves (24, 25). Red-fleshed apples are rich in a variety of active substances such as polysaccharides, polyphenols, anthocyanins, and flavonoids. Notably, the polyphenol content is almost three times that of white-fleshed apples (26). Flavonoids account for a relatively high proportion of the polyphenols in red-fleshed apples, and play a key role in anti-oxidation and maintaining cellular activity (27). Previous studies have investigated anthocyanins, which are a class of flavonoids, in red-fleshed apples. For example, Xu et al. found that red-fleshed apple anthocyanin extract (RAAE) alleviates busulfan-induced dysfunction of the male reproductive system in mice (28) by enhancing sperm motility, increasing the number of mice, and restoring spermatogenesis in male mice. Wang et al. further analyzed the mechanism by which RAAE alleviates busulfan-induced reproductive system dysfunction in mice (29), and found that the total antioxidant capacity (T-AOC), SOD activity, and glutathione catalase activity were significantly increased in the RAAE treatment group, indicating that RAAE can reduce the oxidative injury of busulfan in mice by reducing the content of reactive oxygen species and restoring the reproductive system function of mice. Previous research has also shown that red-fleshed apple flavonoid extract (RAFE) has antioxidant and cell viability maintenance capabilities; however, little research has explored the effects of red-fleshed apple flavonoids on CCl_4_-induced liver injury in mice.

Flavonoids are a class of polyphenolic compounds with a structure of 2-phenylchromone, which has the basic structure of C6-C3-C6. Flavonoids are also important secondary metabolites in plants (30–32) that are widely distributed and present at high levels. In plants, flavonoids are often combined with sugars and exist in the form of aglycones or carbon sugars. Multiple categories of flavonoids exist in plants, primarily chalcones, anthocyanins, flavanones, isoflavonoids, flavonoids, and flavonols (33). Chalcones are unique to apples (34). Years of research have shown that flavonoids have important biological activities that play an important role in plant protection and stress resistance, as well as certain antioxidant and antibacterial properties (30, 35, 36). Flavonoids can reduce the level of oxidative stress and have a certain ability to scavenge free radicals. More importantly, flavonoids can scavenge reactive oxygen species, thereby protecting human health (36, 37). For example, Duangjai extracted flavonoids from Pigeon Pea [*Cajanus cajan* (L.) Millsp.] seeds and explored their antioxidant capacity (38). Their results showed that the flavonoid extract can act as an activator and protect the cell membrane of the cell longevity protein (SIR2/SIRT1) from oxidative stress. Therefore, it is important to explore the further effects of flavonoids on hepatic oxidative stress.

In this study, we establish a CCl_4_-induced mouse liver injury model to explore the alleviation effect and mechanism of RAFE on CCl_4_-induced liver injury in mice. We had previously compared four varieties of red-fleshed apples and found that variety ‘XJ4’ had the highest total phenol and anthocyanin contents with a strong ability to scavenge free radicals (27). At the same time, we previously treated mice with busulfan-induced reproductive injury with ‘XJ4’ anthocyanin extract, and the combined analysis of microbiota and plasma metabolites showed that ‘XJ4’ anthocyanin extract could reduce busulfan-induced reproductive injury (29). So we selected ‘XJ4’ as the experimental material, and the control was the white-fleshed apple ‘Fuji’. The results of this study are expected to provide a theoretical basis for the selection of red-fleshed apples and their nutritional and healthcare functions.

## 2. Materials and methods

### 2.1. Flavonoid preparation and purification

Experimental materials were obtained from ‘XJ4’ apple fruits grown in the Modern Agricultural Science and Technology Demonstration Park of Qingdao Agricultural University, Jiaozhou, China (36°16′49′′N, 120°00′36′′E).

The preparation method of the RAFE was a slightly modified version of the method of Xiang (39). The apple fruits were cleaned, and 0.1% phosphoric acid/ethanol was added as the extracting solution in liquid nitrogen grinding, with a material-to-liquid ratio of 1:30 (g ml^–1^). The mixture was then sonicated (Ultrasonic cleaner, Baoding Shengfeng Instrument Technology Co., LTD., HP) for 30 min and macerated for 12 h with protection from light, then finally filtered to obtain a crude extract solution of flavonoids. Concentration was conducted with a rotary evaporator (Rotary evaporators, Shanghai Yarong Biochemical Instrument Factory, RE-6000A) at 35°C and low speed to remove the organic solvent from the extract until the crude extract became viscous. Finally, the crude extract was diluted by adding an appropriate amount of deionized water (100 ml).

**FIGURE 1:**
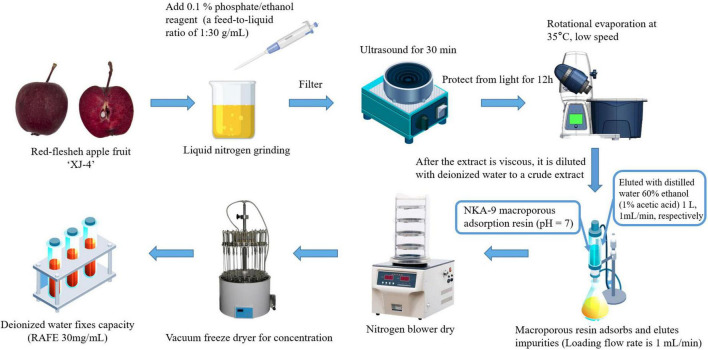
Process flow chart of ‘XJ4’ RAFE preparation and purification.

The flavonoid extract was added to NKA-9 macroporous sorbent resin at pH7 and the flow rate of the upper sample was controlled at 1 ml min^–1^. After reaching equilibrium of resin adsorption, the impurities were eluted with deionized water, the macroporous resin was eluted with 100% (V/V) ethanol eluate at the same flow rate, and all eluates were collected. The eluate was concentrated and dried using a nitrogen blowing machine and vacuum freeze dryer sequentially, and the volume was fixed at a final concentration of 30 mg ml^–1^. Figure 1 shows the process flow for the preparation and purification of RAFE.

### 2.2. Flavonoid HPLC analysis

Protocatechin, catechin, epicatechin, rhizobioside, quercetin, centaureidin-3-O-galactoside, and centaureidin-3-O-glucoside standards were purchased from Sigma-Aldrich (St. Louis, MO, USA). Chromatography-grade methanol was purchased from Tedia (USA). All flavonoid standard stock solutions (1 mg ml^–1^) were stored at a low temperature and protected from light, and the standard curve was constructed using the external standard method. High-performance liquid chromatography (HPLC, USA, Agilent, 1260) was performed under the following conditions: the chromatographic column was an XDB-C18 column (150 × 4.6 mm, 5 μm); the mobile phase A was 1% aqueous acetic acid solution and the mobile phase B was methanol solution; the elution program was 0–10 min, 5–30%, 10–25 min, 30–50%, 25–35 min, 50–70%, and 35–40 min, 70–5% (all mobile phase B); the flow rate was 0.8 ml/min; the sample volume was 20 μl; the column temperature was 30°C; and the wavelength was 280 nm. Qualitative and quantitative analyses of flavonoids in ‘XJ4’ RAFE were performed according to the above methods and conditions.

### 2.3. Animal study

All animal experimental procedures were conducted according to the protocol of the Animal Care and Use Committee of Qingdao Agricultural University (license number: SYXK [SD] 20170005). Specific Pathogen Free (SPF) grade male ICR mice (3 weeks old, 20.0 ± 2.0 g) were housed in an SPF chamber at 22 ± 2°C with a 12 h light/dark cycle and 50–70% humidity, and all mice were fed and watered *ad libitum*. After 3 days of adaptation to the living environment, the mice were randomly divided into the following six groups (10 mice per group): normal control (NC), pathological control (MC), positive control (PC), low RAFE concentration (L-RAFE), medium RAFE concentration (M-RAFE), and high RAFE concentration (H-RAFE). The prepared RAFE was diluted into different concentrations and fed to mice in the RAFE intervention groups by gavage (0.2 mg kg^–1^, L-RAFE; 1 mg kg^–1^, M-RAFE; and 5 mg kg^–1^, H-RAFE). The NC group was administered saline gavage, the MC group was administered saline and CCl_4_ as a model (0.1% CCl_4_ olive oil solution 10 ml kg^–1^, intraperitoneal injection, twice a week, 4 weeks in total), and the PC group was treated with bifenthrin drops in aqueous solution (1 mg kg^–1^, gavage). CCl_4_ was injected intraperitoneally at the same time as the gavage in all groups except the NC group, and the injection volume was the same as that of the MC group. The gavage volume was 0.1 ml mouse^–1^, which was administered for 4 weeks. The establishment of the CCL_4_ liver injury model was established with reference to Keshavarz et al. and slightly modified (40, 41).

### 2.4. Sample collection

After 35 days, the mice were deprived of food for 12 h then euthanized by cervical dislocation under ether anesthesia. After euthanasia, heart blood and liver tissues were taken from the mice, weighed, and stored at −80°C for further analysis.

### 2.5. Biochemical assays of mice plasma and liver tissue

Superoxide dismutase, T-AOC, and MDA diagnostic kits were purchased from Solebro Biotechnology (Beijing, China). AST, ALT, alkaline phosphatase (ALP), and total glutathione (T-GSH) diagnostic kits were purchased from the Nanjing Jiancheng Institute of Biological Engineering, Nanjing, China. Tests were performed according to the manufacturer’s instructions.

### 2.6. Histopathological observation of mice livers

We processed the mice liver tissue samples fixed in 4% paraformaldehyde. The samples were paraffin-embedded and cut into sections 7–8 μm thick, which were stained with hematoxylin-eosin (H&E) dye, then observed and imaged using an optical microscope (Leica fluorescence microscope, Germany Leica Microsystems, DM2500) with a 40× objective to assess pathological changes in the liver tissue.

### 2.7. Sequencing of microbiota from small intestine digesta samples

The samples used for 16S rRNA gene sequencing were fecal samples from mice fed for 5 weeks. Genomic DNA was extracted using the CTAB method (Nobleryder, China), followed by agarose gel electrophoresis to determine DNA purity and concentration. Finally, the DNA sample was diluted with sterile water to 1 ng μl^–1^.

The template was diluted genomic DNA, and the variable region V4 of the 16S rRNA gene consisted of forward primers 515 F (5′-GTGCCAGCMGCCGCGGTAA) and 806 R (3′- GGACTACHVGGGTWTCTAAT). PCR uses specific primers with barcodes and a highly efficient High-Fidelity DNA polymerase [Phusion^®^ High-Fidelity DNA polymerase (M0530S, New England Biolabs, USA)] to ensure amplification efficiency and accuracy. The PCR system comprised Phusion Master Mix (2×) 15 μl, PrimerF (1 μM) 1 μl (1 μM), PrimerR (1 μM) 1 μl (1 μM), gDNA (1 ng/μl) 10 μl (5–10 ng), and ddH_2_O to make up 30 μl of the system. The PCR reaction program was: 98°C pre-denaturation for 1 min; 30 cycles of 98°C for 10 s, 50°C for 30 s, 72°C for 30 s, and 72°C for 5 min.

PCR products were detected by electrophoresis on 2% agarose gel. The PCR products that passed the test were purified using magnetic beads and quantified using enzyme labeling, then detected by 2% agarose gel electrophoresis after mixing the product in equal amounts according to the concentration of the product. Target bands were recovered using a gel recovery kit (Qiagen). Library construction was performed using a library construction kit (TruSeq DNA PCR-Free Sample Preparation Kit). The constructed libraries were then quantified by Qubit and Q-PCR. If the library qualified, sequencing was performed using NovaSeq6000 (Illumina Novaseq6000, Illumina Corporation, Illumina, San Diego, CA, USA).

### 2.8. Statistical analysis

Data analysis of the gut microbes was performed using the cloud platform.^1^ If there were only two groups of samples, the *t*-test and Wilcoxon test were used; if there were more than two groups of samples, the Tukey test and Kruskal–Wallis test were used. The remaining graphs were produced using GraphPad Prism software (version 8.0.2; GraphPad Software Inc., San Diego, CA, USA). The data were tested for multiple comparisons using Dunnett’s method in SPSS (version 24.0; SPSS Inc., Chicago, IL, USA) and ordinary one-way ANOVA. P < 0.05 was set as the threshold to indicate a significant difference.

### 2.9. Data and materials availability

The microbiota raw sequencing data generated in this study have been uploaded to the NCBI SRA database with accession numbers: RJNA901622.^2^

## 3. Results

### 3.1. HPLC analysis of RAFE flavonoid composition and contents

**FIGURE 2:**
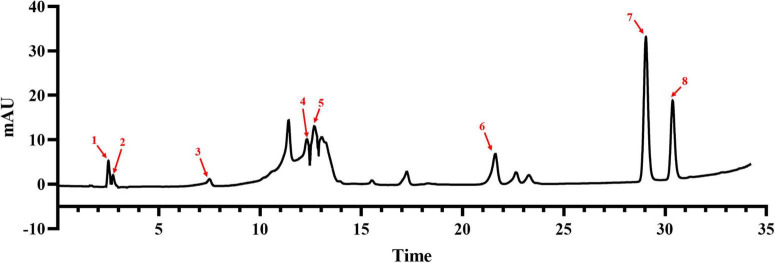
Composition and content of flavonoids in RAFE based on HPLC analysis. HPLC plot of RAFE, where the eight peaks labeled in the plot correspond to the flavonoid compounds with the same serial numbers as those in Table 1.

To clarify the composition and content of flavonoids in ‘XJ4’ RAFE, we used HPLC to draw standard curves for eight standards, such as catechins, and delineated the linear range of each standard (Table 1). Flavonoids were identified by comparing the retention times of sample peaks and standard peaks, and the contents of various flavonoids in RAFE were calculated using the standard curves (Figure 2). In the chromatogram of ‘XJ4’ RAFE (Figure 2), we detected eight flavonoid compounds, including one flavonol, three flavanols, two anthocyanins, and two dihydrochalcone. The data showed that ‘XJ4’ RAFE had a high content of catechin (49.346 mg⋅100 g^–1^), followed by quercetin (23.087 mg⋅100 g^–1^), and the least abundant substance was rhizobioside (0.827 mg⋅100 g^–1^). Interestingly, the levels of centaureidin-3-O-galactoside and centaureidin-3-O-glucoside reached 13.306 mg⋅100 g^–1^ and 4.426 mg⋅100 g^–1^, respectively (Table 1).

**TABLE 1:** Contents of flavonoids identified in RAFE and related HPLC data.

Peak number	Compound	Calibration curves	*R* ^2^	Test range (μg/ml)	Retention time (min)	Content (mg/100 g)
1	Cyanidin-3-O-galactoside	*y* = 20569x−226.41	0.9907	6.25–100.0	2.504	13.306
2	Cyanidin 3-O-glucoside	*y* = 4343.9x+3.7214	0.9996	6.25–100.0	2.670	4.426
3	(−)-Catechin hydrate	*y* = 5350.1x−16.872	0.9977	0.25–8.0	7.452	5.148
4	(+)-Catechin	*y* = 6171.9x+8.7721	0.9986	3.75–60.0	12.304	49.346
5	(−)-Epicatechin	*y* = 15833x−20.699	0.9931	3.75–60.0	12.674	19.719
6	Phloridzin	*y* = 16967x−1.0121	0.9995	6.25–100.0	21.610	0.827
7	Quercetin	*y* = 32111x−254.54	0.9981	1.56–50.0	29.048	23.087
8	Phloretin	*y* = 121700x+13.2	0.9982	6.25–100.0	30.370	1.923

### 3.2. Effects of RAFE on body weight and liver of mice with CCl_4_-induced liver injury

**FIGURE 3:**
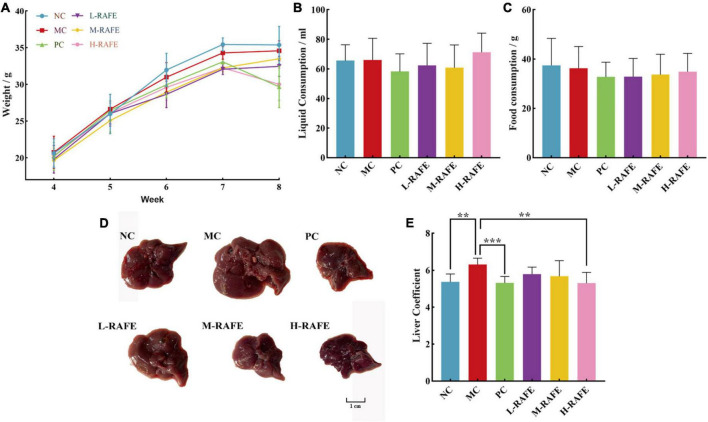
Effects of RAFE on basic life indicators and livers of mice. **(A)** Weekly body weight, **(B)** weekly fluid consumption, **(C)** weekly food consumption, **(D)** morphological observation, and **(E)** liver coefficient. Data are expressed as the mean ± SD (*n* = 4 or 6) **P < 0.01, ***P < 0.001, compared with the pathology group.

Studies have shown that flavonoids have protective effects against CCl_4_-induced liver injury in mice. An elevated liver index is an important indicator of liver injury. During rearing, body weight, fluid consumption, and food consumption were measured every 2 days in each group. Based on the weekly data, we found no significant changes (*P* > 0.05) in the above three basic life indicators (Figures 3A–C). CCl_4_-treated mice showed a significant increase in liver size and liver index compared to the normal controls, but RAFE treatment produced a certain recovery effect, especially in the H-RAFE group, where the liver coefficient was significantly lower than that of the MC group (*P* < 0.01) and almost returned to the level of the NC group (Figures 3D, E). The PC group showed a significant effect on the recovery of liver coefficients (Figure 3E). According to the liver images, the livers of the MC group exhibited obvious edema expansion and a yellowish color, as well as many white dotted lesions on the surface of the liver. Conversely, treatment with three different concentrations of RAFE and bifendatatum restored the color and edema of the liver, especially in the M-RAFE, H-RAFE, and PC groups, which were almost restored to normal levels (Figure 3D).

### 3.3. Effect of RAFE on serum biochemical indexes of mice with CCl_4_-induced liver injury

**FIGURE 4:**
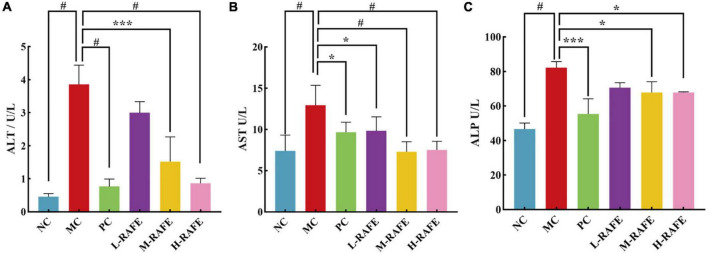
Effect of RAFE on serum biochemical parameters in mice. **(A)** ALT, **(B)** AST, and **(C)** ALP. Data are expressed as the mean ± SD (*n* = 4 or 6) *P < 0.05, ***P < 0.001, ^#^*P* < 0.0001, compared with the pathological group.

Liver injury adversely affects the transport function and membrane permeability of hepatocytes and causes enzymes to leak out of cells. The most important enzymes that reflect liver injury are ALT, AST, and ALP. In this study, we determined the enzymatic activity levels of ALT, AST, and ALP in mouse plasma (Figure 4). We found that ALT, AST, and ALP enzyme activities increased sharply after CCl_4_ intervention in plasma, to 3.86, 12.93, and 82.16 U/L, respectively, whereas the enzyme activity of NC group was only 0.46, 7.38, and 46.56 U/L (Figure 4). This indicated that the liver tissue of mice was severely damaged after CCl_4_ treatment. After RAFE intervention, the levels of ALT, AST, and ALP were all significantly reduced, especially in H-RAFE and M-RAFE groups, where the enzymatic activity of AST was restored to almost normal levels (*P* < 0.0001) (Figure 4B). Simultaneously, ALP and ALT levels were significantly decreased in the H-RAFE and M-RAFE groups. Interestingly, RAFE reduced ALT activity in a dose-dependent manner (Figure 4A). Bifendate, a clinical drug used for the treatment of hepatitis, also significantly reduced the plasma levels of ALT, AST, and ALP (Figure 4). These results suggest that RAFE can effectively relieve liver injury caused by CCl_4_.

### 3.4. Effects of RAFE on biochemical indices and liver in CCl_4_-liver injury mice

**FIGURE 5:**
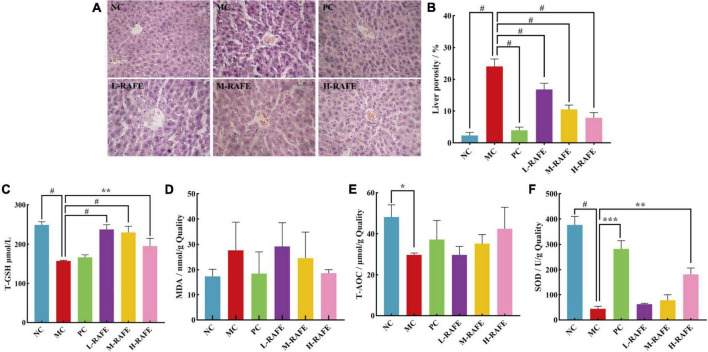
Effect of RAFE on biochemical indexes of mouse livers and liver histology. **(A)** Histopathological observations of mouse livers (40× magnification), **(B)** liver porosity, **(C)** T-GSH, **(D)** MDA, **(E)** T-AOC, and **(F)** SOD. Data are expressed as the mean ± SD (*n* = 4 or 6) *P < 0.05, **P < 0.01, ***P < 0.001, ^#^*P* < 0.0001, compared with the pathological group.

Carbon tetrachloride-induced liver injury is associated with lipid peroxidation in mice, and elevated MDA levels are a key manifestation of liver injury caused by lipid peroxidation. In this study, the level of MDA increased from 17.36 nmol/g quality in the MC group to 27.65 nmol/g quality in the NC group (Figure 5D), which indicated that CCl_4_ treatment caused significant peroxidative injury in the mouse liver. However, the level of MDA decreased after RAFE treatment, and the most pronounced effect was in the H-RAFE group, with a reduction of 18.60 nmol/g quality (Figure 5D). This suggests that RAFE alleviates CCl_4_-induced liver peroxidation. SOD is the most important antioxidant enzyme in biological systems. T-GSH is the main non-enzymatic antioxidant and regulator of intracellular redox homeostasis. Normally, there is a physiological balance between the production of reactive oxygen species, free radicals, and antioxidant enzymes. Peroxidation of liver tissues usually results in high consumption of antioxidant enzymes/antioxidants, such as SOD and T-GSH. In this study, SOD, T-GSH, and T-AOC decreased to 44.80 U/g quality, 157.59 μmol/L, and 29.76 μmol/g quality, respectively, in the MC group (Figures 5C, E, F). However, RAFE intervention significantly increased the levels of SOD, T-GSH, and T-AOC (Figures 5C, E, F), which followed the same trend as that of enzyme activity in the mouse plasma (Figure 4). Interestingly, the best recovery of T-GSH was observed in the L-RAFE group, which returned to normal levels of 237.67 μmol/L (Figure 5C). The PC group also showed better recovery of biochemical parameters in the liver (Figures 5C–F). Recovery of these liver indices showed that RAFE can improve CCl_4_-induced oxidative stress and help reduce oxidative injury in the mouse liver.

Histopathological observations provide visual data on the recovery effect of RAFE in mouse liver injury. H&E staining of liver sections showed that the NC group had well-preserved cytoplasm and typical hepatocytes with clearly visible central veins. The hepatocytes were tightly bound to each other, and the liver porosity was only 2.34% (Figures 5A, B). In contrast, significant pathological changes were observed in the hepatocytes around the central vein of the liver in the MC group, and the cells were loosely bound with a liver porosity of 24.05%. However, after RAFE intervention, hepatic tissue cells normalized, liver porosity significantly decreased, hepatocellular lesions around the central vein were reduced, and the H-RAFE group showed the best recovery, with a 7.89% reduction in porosity, indicating a recovery effect that was second only to that of bifendate. The results of the histological observation analysis were consistent with the trend of serum and liver recovery, which suggested that RAFE intervention significantly ameliorates severe histological lesions in mice with CCl_4_-induced liver injury.

### 3.5. Effect of RAFE on intestinal microorganisms in mice with CCl_4_-induced liver injury

**FIGURE 6:**
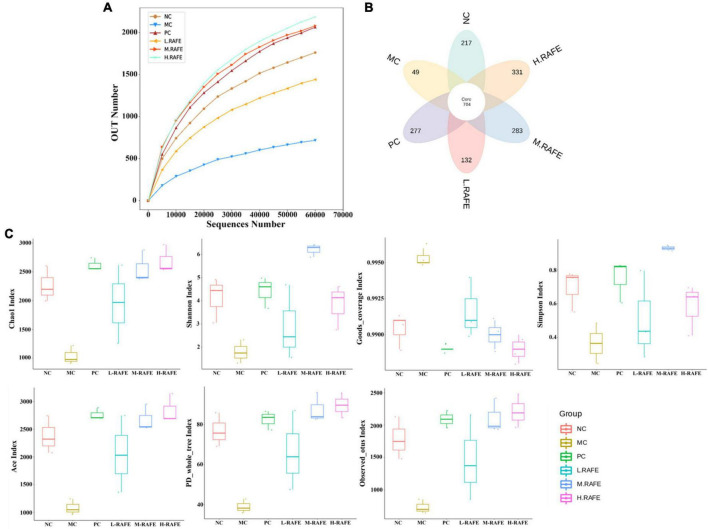
Effect of RAFE on OTUs and alpha diversity of mice intestinal microorganisms. **(A)** Grouped dilution curves, where a flatter curve indicates a more uniform species distribution, **(B)** petal plot, and **(C)** alpha diversity analysis (Chao1, Shannon, Goods, Simpson, Ace, PD, and observed). *n* = 3 samples/group.

It has been shown that there is a bidirectional relationship between the liver and gut microbes, with the portal vein enabling direct transport of gut-derived products to the liver, as well as a hepatic feedback pathway of bile and antibody secretion to the intestine (42). Here, we investigated whether flavonoids extracted from red-fleshed apples can restore liver injury by regulating intestinal microorganisms. We determined the effect of RAFE on the overall structure and abundance of mouse intestinal microorganisms by analyzing the gene sequence of the 16S rRNA (V4 region) in mouse feces. The smoothed dilution curve indicated the reliability of the sequencing results (Figure 6A). The petal diagram showed that CCl_4_ reduced the number of operational taxonomic units (OTUs) in the pathological group from that in the normal control group, whereas the bifendate and RAFE intervention groups both restored the number of similar microorganisms to a certain extent, especially in the H-RAFE group. This suggests that RAFE has the potential to restore intestinal microbes (Figure 6B). The statistical results of the alpha index showed that the diversity of NC and MC groups was negatively correlated, and intervention with RAFE partially restored the diversity of intestinal microbes in mice with CCl_4_-induced liver injury. Overall, H-RAFE intervention showed the best effect; however, drug treatment also restored intestinal microbe levels (Figure 6C).

**FIGURE 7:**
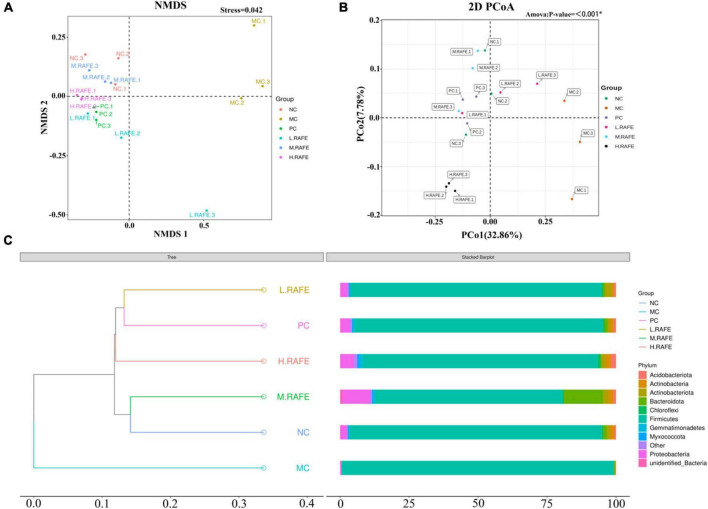
Effect of RAFE on beta diversity of mice intestinal microorganisms. **(A)** Non-metric multidimensional scaling analysis based on OTU levels, **(B)** 2D plot of unweighted UniFrac distance-based principal co-ordinates analysis, where closer sample distances indicate more similar species composition structures, and **(C)** unweighted pair-group method with arithmetic mean clustering tree based on unweighted UniFrac distances. *n* = 3 samples/group.

Beta diversity analysis was used to assess the similarity between groups. OTU-based non-metric multidimensional scaling analysis showed that the MC group was clearly distinct from the other treatment groups, whereas all other treatment groups had certain similarities with the NC group, which suggested that RAFE could restore the composition of intestinal microbes (Figure 7A). We then performed principal co-ordinates analysis based on the unweighted UniFrac distance. The results showed that the MC group had lower community similarity with the NC group, whereas the distance to the NC group after RAFE treatment was relatively close and the species composition structure was similar, illustrating the success of CCl_4_ modeling and the therapeutic effect of RAFE (Figure 7B). The unweighted pair-group method with arithmetic mean dendrogram based on the unweighted UniFrac distance showed that the NC and MC groups had different gut microbial compositions because the two groups were located on different branches, and the microbial composition after RAFE and bifendate intervention was closer to that of the NC group, especially for the M-RAFE group (Figure 7C).

**FIGURE 8:**
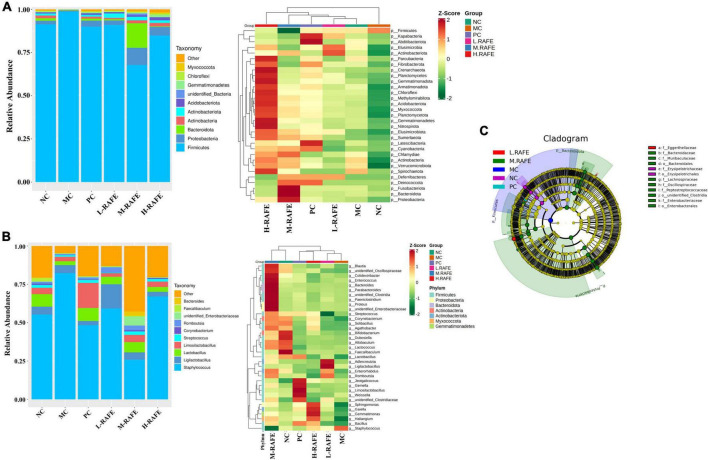
Effect of RAFE on species abundance at different taxonomic levels of mice intestinal microorganisms and linear discriminant analysis effect size. **(A)** Abundance chart at the gate level, with a stacked bar diagram of relative species abundance at the gate level on the left and a heat map of species abundance at the gate level for the top 35 microorganisms on the right, **(B)** abundance chart at the genus level, with a stacked bar diagram of relative species abundance at the genus level on the left and a heat map of species abundance at the genus level for the top 35 microorganisms on the right, and **(C)** evolutionary branching diagram, where the circles radiating from interior to exterior represent the taxonomic level from phylum to genus or species. Each small circle at different taxonomic levels represents a taxon at that level, and the diameter of the small circles is proportional to the relative abundance size. Coloring principle: species with no significant differences are uniformly colored yellow, and species with differences follow the group for coloring. *n* = 3 samples/group.

To identify the specific taxa associated with RAFE, the relative abundance of intestinal microbes was determined at the phylum and genus levels (Figures 8A, B). At the phylum level, the microbial community was mainly composed of Firmicutes, Proteobacteria, and Bacteroidetes. The abundance of Firmicutes was higher in the MC group, whereas the abundance of Bacteroidetes was lower, resulting in an increase in the ratio of Firmicutes to Bacteroidetes (F/B) compared to the NC group. RAFE treatment affected the F/B ratio in two ways, increasing the relative abundance of Bacteroidetes and decreasing the relative abundance of Firmicutes, thereby reducing the F/B ratio and returning F/B to normal levels. Interestingly, the interventions of both RAFE and bifendate increased the relative abundance of Proteobacteria and Actinobacteria compared to that in the MC group (Figure 8A). At the genus level, the intervention of RAFE reduced the relative abundance of *Staphylococcus*, increased the relative abundance of probiotics such as Lactobacillus, and restored the intestinal microbial level almost back to normal (Figure 8B). To further validate the differences in intestinal microbes across all groups, the abundance of different species was analyzed at the phylum and genus levels. On the phylum-level abundance heat map, the top 35 dominant bacterial groups in the NC group were similar to those in the bifendate group and RAFE intervention groups (Figure 8A). Interestingly, on the genus-level heatmap, the dominant flora of the NC group was only similar to that of M-RAFE (Figure 8B).

Linear discriminant analysis effect size indicated that the dominant microbes in the MC group were Firmicutes, and intervention with M-RAFE mainly increased nine microbiota induced by CCl_4_ in mice, including *Lachnospiraceae*, *Muribaculaceae*, *Clostridia*, and other probiotics (Figure 8C). Taken together, these data suggest that the gut microbiota of mice induced by CCl_4_ are imbalanced, and that RAFE can modulate gut disturbance in mice with liver injury by increasing the microbial diversity and relative abundance of probiotics.

## 4. Discussion

Liver disease is a deadly disease that occurs worldwide. The abuse of alcohol, chemical drugs, and food safety problems lead to the frequent occurrence of liver injury. According to statistics, more than 75 million people worldwide are at risk of alcoholic liver injury and related diseases. Moreover, approximately 2 billion people are obese and overweight so at risk of non-alcoholic fatty liver disease (43). The current common treatment for liver injury is drug intervention, which can cause undesirable side effects. In recent years, natural plant extracts have received extensive attention because of their safety and ease of preparation. Natural plant extracts are rich in various components, such as flavonoids, which are widely recognized as effective functional components.

Flavonoids have extremely high medicinal value as natural plant extracts, and have been shown to prevent and treat cardiovascular diseases by reducing blood lipids and cholesterol, exhibit anti-oxidation effects, and protect the liver (44, 45). Studies have shown that the bioactive components in blueberries include anthocyanins, polyphenols, and pectin, with the pectin in blueberry powder also including anthocyanin-3-glucoside and anthocyanin. These components can protect vision by reducing vascular endothelial growth factors and activating Akt signaling (46). Ma et al. found that silymarin intervention improved liver steatosis and hepatic lobular inflammation (46). Another study found that black tea extract prevents liver fibrosis in rats by regulating the TGF-β/Smad/ERK signaling pathway (47). Thus, flavonoids have high medicinal value. Nevertheless, few studies have analyzed apple flavonoids, and none have used RAFE to study liver injury and intestinal microbes in CCl_4_-induced mice. The flavonoid content varied significantly among apple varieties (48), and the flavonoids in the peel and flesh of common apples on the market varied greatly (49), but the composition of flavonoids in apples did not vary much. Our XJ4, as a red-fleshed apple, is rich in flavonoids in both skin and flesh. Compared with the common apples in the market, the flavonoid content is much richer. In this study, we showed that RAFE contains eight flavonoids, including one flavonol, three flavanols, two anthocyanins, and two dihydrochalcones. Therefore, we speculate that RAFE can improve antioxidant capacity and relieve liver injury by regulating intestinal microbes to interfere with biochemical indexes of the plasma and liver.

The CCl_4_-induced liver injury model is widely recognized as a common model for screening liver-protective drugs. This model can lead to increased liver weight, an abnormal increase in serum ALT and AST levels, a significant increase in SOD activity, and decreased levels of GSH (50). CCl_4_ can also cause lesions in liver tissue, resulting in loose liver tissue, loss of structural integrity, gaps around liver cells, and inflammatory reactions (43). Studies have shown that the flavonoid extract of baicalin can alleviate abnormalities in serum biochemical indicators and heterologous liver injury by reducing inflammation and oxidative injury (51, 52). Another study showed that puerarin alleviates liver injury in rats and zebrafish and attenuates inflammatory infiltration (52, 53). The liver index directly reflects changes in liver weight, and an abnormal liver index is a clear sign of the development of liver disease. Yan et al. found that lychee extract, which is rich in flavonoids, attenuates CCl_4_-induced hepatomegaly in rats and has a positive effect on pathological changes in liver tissue (54). Our study found that RAFE has a similar function, not only normalizing the liver index and restoring abnormal enzyme activity and biochemical indices in serum, but also relieving liver tissue injury and reducing liver porosity. In addition, MDA and T-AOC in the liver showed that CCl_4_ also caused oxidative stress and oxidative injury to the liver. Interestingly, oxidative injury to the liver was alleviated by different doses of RAFE, where the degree of remission was positively correlated with the dose. This shows that RAFE has antioxidant functions and alleviates liver injury; however, the specific mechanism remains unclear.

The gut has a complex microbial system for nutrient absorption, metabolism, and protection (55). Some of these colonies, such as *Lachnospiraceae*, *Lactobacillus acidophilus*, and *Clostridium*, are considered beneficial, whereas other bacterial groups, such as *Pseudomonas*, *Staphylococcus*, and *Fusobacterium*, are pathogenic bacteria that may produce toxins (56). Poor living and eating habits and the exogenous intake of harmful substances will cause imbalances in the abundance of intestinal microorganisms, as well as structural changes, increase the toxins produced by harmful bacterial colonies, or fail to maintain the corresponding functions of beneficial bacteria, resulting in diarrhea and injury to internal organs. As the largest digestive gland in the human body, the liver is one of the most vulnerable organs (57, 58). It has been shown that there is a bidirectional relationship between the liver and the gut and its microbiota – the gut-liver axis – and that toxic substances can disrupt gut microbes and the intestinal barrier, thereby increasing microbial exposure and the pro-inflammatory environment of the liver (42). In our previous study, we showed that RAAE could improve the abundance and structure of gut microbes. Therefore, we speculate that RAFE may also have beneficial effects on liver injury by improving the diversity and abundance of gut microbial populations. Studies have shown that aronia polyphenols can protect the liver by maintaining the balance of intestinal flora and reducing the translocation of bacteria to enhance intestinal barrier function (57). Changes in the gut microbiota driven by blueberry extract reduce inflammation, protect the integrity of the gut epithelium, and improve gut health, thereby reducing the translocation of bacterial products across the epithelial barrier (59). Li et al. found that treatment of mice on a high-fat diet with sarsaparilla polyphenols increased the ratio of Bacteroidetes to Firmicutes in their gut flora, but decreased the relative abundance of Verrucobacterium, thereby alleviating non-alcoholic liver injury caused by a high-fat diet (60). Our study showed that RAFE can interfere with the gut microbiome of mice, which is consistent with other related studies. However, we found that the regulation of gut microbes by RAFE, especially the regulation of the F/B ratio, not only decreased or increased the relative abundance of a certain phylum but also increased Bacteroidetes and decreased the relative abundance of Firmicutes, resulting in a change in the ratio of the two bacterial groups. Moreover, M-RAFE intervention mainly increased the nine species of microflora induced by CCl_4_ in mice, including probiotics such as *Lachnospiraceae*, *Muribaculaceae*, and *Clostridia*. This phenomenon has rarely been reported in previous studies.

## 5. Conclusion

In conclusion, our study shows that RAFE contains a variety of flavonoids that can restore the disturbance of intestinal microbes by regulating beneficial bacteria and reducing harmful bacteria. RAFE also improves the abnormal biochemical indicators of the plasma and liver caused by CCl_4_ poisoning, relieves oxidative injury to liver tissue, and reduces inflammation to protect the liver. According to experiments at different RAFE concentrations, we found that the relieving effect of RAFE on liver injury was not dose-dependent. This suggests that RAFE could be used for the adjuvant therapy and prevention of liver injury. Therefore, this research can promote the use of RAFE in food and medical applications.

## Data availability statement

The datasets presented in this study can be found in online repositories. The names of the repository/repositories and accession number(s) can be found in the article/supplementary material.

## Ethics statement

This animal study was reviewed and approved by the Animal Care and Use Committee of Qingdao Agricultural University (license number: SYXK [SD] 20170005).

## Author contributions

YC and YW designed the research, performed the experiments, and wrote the manuscript. SJ and JX made contributions to perform the experiments and analysis the data. BW provided experiments assistance to YC. XS provided the suggestions and made contributions to design of the experiment. XS and YZ designed this experiment and revised the manuscript. All authors contributed to the article and approved the submitted version.

## Footnotes

1
https://cloud.metware.cn

2
https://www.ncbi.nlm.nih.gov/sra
